# Assessment of Anti-TNF-α Activities in Keratinocytes Expressing Inducible TNF- α: A Novel Tool for Anti-TNF-α Drug Screening

**DOI:** 10.1371/journal.pone.0159151

**Published:** 2016-07-14

**Authors:** Sutthirat Udommethaporn, Tewin Tencomnao, Eileen M. McGowan, Viroj Boonyaratanakornkit

**Affiliations:** 1 Graduate Program in Clinical Biochemistry and Molecular Medicine, Faculty of Allied Health Sciences, Chulalongkorn University, Bangkok 10330, Thailand; 2 Department of Clinical Chemistry, Faculty of Allied Health Sciences, Chulalongkorn University, Bangkok 10330, Thailand; 3 Chronic Disease Solutions Team, School of Life Sciences, University of Technology Sydney, Ultimo, 2007, Sydney, Australia; Florida International University, UNITED STATES

## Abstract

Tumor necrosis factor alpha (TNF-α) is a pro-inflammatory cytokine important in normal and pathological biological processes. Newly synthesized pro-TNF-α is expressed on the plasma membrane and cleaved to release soluble TNF-α protein: both are biologically active. Secreted TNF-α signals through TNF receptors and the membrane-bound TNF-α acts by cell contact-dependent signaling. Anti-TNF-α antibodies have been used effectively for treatment of chronic inflammation, however with adverse side effects. Thus, there is a need for new anti-TNF-α small molecule compounds. Anti-TNF-α activity assays involve treatment of keratinocytes with exogenous TNF-α before or after anti-TNF-α incubation. However, this model fails to address the dual signaling of TNF-α. Here we describe a Doxycycline (Dox)-inducible TNF-α (HaCaT-TNF-α) expression system in keratinocytes. Using this *in-vitro* model, we show cell inhibition and induced expression of pro-inflammatory cytokines and markers, including IL-1β, IL-6, IL-8, NF-κB1, and KRT-16, similar to cells treated with exogenous TNF-α. Sufficient secreted TNF-α produced also activated IL-1β and IL-8 expression in wt HaCaT cells. Importantly, stimulated expression of IL-1β and IL-8 in HaCaT-TNF-α were blocked by Quercetin, a flavanol shown to possess anti-TNF-α activities. This novel *in vitro* cell model provides an efficient tool to investigate the dual signaling of TNF-α. Importantly, this model provides an effective, fast, and simple screening for compounds with anti-TNF-α activities for chronic inflammatory disease therapies.

## Introduction

Inflammation is an essential innate immunity response that is crucial to combat pathogens. However, dysregulated and untimely inflammation contributes to several chronic inflammatory diseases such as psoriasis, atopic dermatitis, rheumatoid arthritis, coronary heart diseases, Crohn’s disease and cancer [1–3]. For example, chronic inflammation due to virus and bacterial infections, such as herpes simplex virus (HSV) as well as *Chlamydia pneumonia*, has been shown to increase coronary heart disease risk [4]. Local inflammation in breast tissues due to obesity has also been reported to increase risk of aggressive breast cancer through increased local estrogen production [5], Given the significance of inflammation in a variety of common diseases, the use of anti-inflammatory drugs are currently being used in therapeutic regimes, especially in cancer [6, 7]. Nevertheless, although it is accepted that inflammation plays a central role in many diseases, its role in disease progression is a poorly understood process.

The inflammation process involves intricate networking and interactions between immune cells and pro-inflammatory mediators such as Interleukin (IL)-1, IL-6, nuclear factor kappa-light chain enhancer of activated B cell (NF-κB) and tumor necrosis factor alpha (TNF-α) [8]. TNF-α is a pro-inflammatory cytokine that plays a key role in cell survival and apoptosis of normal and malignant cells [9, 10]. This pro-inflammatory cytokine is expressed in several cell types including activated macrophages, T cells, natural killer cells, dendritic cells, mast cells and keratinocytes [11]. TNF-α is one of the key pro-inflammatory mediators involved in the pathogenesis of psoriasis, atopic dermatitis and rheumatoid arthritis and is often found in high amounts around psoriasis skin lesions [12].

Pathophysiological conditions of psoriasis involves hyperproliferation of keratinocytes in combination with abnormal differentiation of the epidermis layer and infiltration of inflammatory cytokines secreted from activated T cells or antigen presenting cells from the dermis and epidermis cell layers. TNF-α is one of the most important molecules involved the induction and progression of psoriasis and stimulates the production of inflammatory cytokines such as IL-6, IL-8 and granulocyte macrophage colony stimulating factor (GM-CSF) resulting in an accumulation of inflammatory cells in the dermis and epidermis layers of psoriatic lesions [13]. Blockage of TNF-α, by anti-TNF-α humanized monoclonal antibodies (Mabs), such as Infliximab Etanercept and Adalimumab, which bind with high affinity to TNF-α, has been shown to successfully treat psoriasis [14]. Patients treated with anti-TNFα Mabs often show reduction in skin and joint inflammation, decreased dermis thickening, and increased keratinocyte differentiation [13]. In addition to psoriasis treatment, anti-TNF-α Mabs have been used for treatment of other immune-mediated inflammatory diseases including rheumatoid arthritis, Crohn’s disease, ankylosing spondylitis, and ulcerative colitis [14]. Conversely, use of anti-TNF-α Mabs can lead to serious adverse side effects, other malignant diseases [15, 16] autoimmune diseases [17, 18] and development of skin lesions or psoriasis [19, 20]. Moreover, a sub-set of patients do not respond to anti-TNF-α therapies or alternatively develop resistant after initial improvement [14].

To minimize immunological and other adverse effects caused by anti-TNF-α Mabs an alternative approach, with potentially fewer immune-mediated side effects, is the use of small chemical molecules acting as TNF-α antagonists by blocking TNF-α activities. These TNF-α antagonists may be used as a complementary treatment for chronic inflammatory diseases. There are several advantages of developing small-molecular TNF-α antagonists including, targeting of specific signaling pathways, high tissue penetration, ease of use and economical manufacturing [21].

Synthesis and secretion of TNF-α involves a two-step process. Newly synthesized pro-TNF-α is expressed on the plasma membrane and cleaved by TNF-α converting enzyme or (TACE or ADAM-17) to release a soluble TNF-α protein [9, 21]. Both membrane-bound and secreted TNF-α are biologically active and there is evidence to suggest membrane-bound TNF-α also plays a role in cell-cell contact mediated juxtacrine cell signaling [21]. Membrane bound TNF-α can transmit signals both as a ligand and as a receptor through cell-cell contacts [22]. However, soluble and membrane TNF-α have been shown to possess different biological activities. Membrane bound TNF-α mediates antimicrobial activity and plays a significant role in the host defence against acute M. tuberculosis infection [23]. A recent study demonstrated that expression of soluble TNF-α promoted tumor growth while membrane-bound TNF-α -expressing tumors had reduced growth and were mostly devoid of infiltrated myeloid cells [24]. Transgenic mice expressing only membrane bound TNF-α, in the absence of soluble TNF-α, exhibited colitis [25], suggesting important physiological role of the dual TNF-α signaling.

However, the common and distinct biological functions of membrane-bound and secreted TNF-α remains to be determined. The current *in vitro* cell-based model used for anti-TNF-α activity screening in keratinocytes (HaCaT cells) involves treating cells with recombinant purified TNF-α before or after treatment with chemical compounds or extracts [26–29]. However, these *in vitro* cell models are limited. In many chronic inflammatory diseases, such as psoriasis, rheumatoid arthritis and inflammatory bowel diseases, cells themselves express both membrane bound and secreted TNF-α, suggesting TNF-α exerts its biological actions in these cells through the dual action of both forms of TNF-α (membrane bound and secreted). Addition of exogenous TNF-α or the secreted form of TNF-α activates TNF-α receptor-mediated signaling, nevertheless there is no evidence to suggest that contact-dependent signaling mediated by membrane bound TNF-α is affected. Therefore, anti-TNF-α activities assayed by current *in vitro* cell models may lack an important signaling component mediated by membrane bound TNF-α.

To provide an alternative and more effective *in vitro* cell-based model for the identification of novel small-molecule TNF-α antagonists, we constructed inducible TNF-α keratinocyte (HaCaT) cell lines that mimic expression of endogenous TNF-α from activated keratinocytes *in vivo*, allowing evaluation of both membrane-bound and secreted components of TNF-α signaling. We show that TNF-α is induced by doxycycline (Dox) in a dose-dependent manner and demonstrate that ectopic expression of TNF-α in keratinocytes produces comparable TNF-α-mediated responses. Importantly, our results demonstrate that these responses are attenuated by a known TNF-α blocker in this newly developed cell model, similar to those of keratinocytes treated with exogenous TNF-α. This new *in vitro* cell model provides an efficient system to explore TNF-α downstream signaling events and inflammatory responses. Importantly it provides a fast and convenient way to screen, identify and evaluate anti-TNF-α small molecules.

## Materials and Methods

### Cell lines and culture

Human embryonic kidney (HEK293T) cells were obtained from American Type Culture Collection (ATCC) and used for lentiviral production. HEK293T were cultured in Dulbecco's modification of Eagle's medium (DMEM; HyClone Laboratories, Logan, USA) supplemented with 10% fetal bovine serum (FBS;Merck Millipore, Darmstadt, Germany) and 1% penicillin streptomycin (PenStrep) (HyClone Laboratories, Logan, USA). HaCaT cells, immortalized human epidermal keratinocytes [30], were purchased from Cell Lines Service (CLS, Heidelberg, Germany) and cultured in DMEM supplemented with 10% FBS and 1% PenStrep. All cells were cultured at 37°C in a humidified atmosphere 5% CO_2_. All cultures were routinely tested and were mycoplasma-free.

### Construction of pHAGE-TNF-α plasmids

To construct the tetracycline (Tet)-inducible vector TNF-α, a pHAGE-TNF-α encoding TNF-α was synthesized. The hTNF-α cDNA was PCR amplified from pMD18-T-hTNF-α cDNA (purchased from Sino Biological Inc., Beijing, China) using a TNF-α specific forward primer (5’-GAT CGC GGC CGC GAC ACC ATG AGC ACT GAA AGC ATG ATC-3’) and a TNF-α specific reverse primer (5’-GAT CGG CGC GCC AGG GCA ATG ATC CCA AAG T-3) containing restriction sites for NotI and AscI respectively. Cycling conditions were as follows: an initial denaturing step (98°C, 3 min), amplification 30 cycles of 45 sec, denaturation at 98°C, 45 sec of annealing at 60°C, 50 sec of extension 72°C and final extension step (72°C, 10 min) using a Thermal Cycler (MJ Research Inc., USA). The PCR products were separated by electrophoresis on a 1% agarose gel and visualized by ethidium bromide staining. The resulting PCR products were further purified using QIAquick gel extraction kit (Qiagen, Cat # 28704) according to the manufacturer's instructions. PCR products were digested with NotI/AscI (Thermo Scientific, NY, USA) and inserted into NotI/AscI digested pENTR/D-TOPO (Invitrogen, USA) to generate pENTR/D-TNF-α. cDNA was then cloned into the attR1 and attR2 sites of pHAGE-Dest, (pINDUCER20, Tet-inducible bicitronic lentiviral vector for inducible expression of the gene of interest driven by the tetracycline response element (TRE)). Constitutive expression of the neomycin resistance gene is driven by the ubiquitin C (Ubc) promotor [31] using the Gateway cloning system with LR Clonase II as suggested by the manufacturer (Invitrogen/Thermo Fisher, USA). The pHAGE-Dest lentiviral vector is a Tet-on vector that encodes a recombinant tetracycline controlled transcription factor (rtTA3) gene [31]. Therefore expression of TNF-α can be induced by increasing the concentration of a tetracycline analog, Dox. Similar cloning strategy using a Green Fluorescence Protein (GFP cDNA) was also used to generate a Tet-inducible pHAGE-GFP lentiviral construct.

### Production of lentiviral particles

To generate lentiviral particles, 6-well tissue culture plates coated with 1 mL of 0.1mg/mL poly-D-lysine solution (Sigma Aldrich, MO, USA) were incubated for least 4 hr at room temperature (RT). Plates were washed four times with 1X Phosphate buffer saline (PBS) (Hyclone, NJ, USA), PBS was removed and plates air-dried under sterile conditions. HEK293T cells were seeded at 1x10^5^ cells in 6-well tissue culture plates to achieve 50–60% confluency per well and incubated for 24 hr. Cells were treated with Opti-MEM I medium (Gibco/Life Technologies, Gaithersburg, USA) mixed with packaging plasmid (psPAX2), envelope plasmid (pMD2G), DNA construct (pHAGE-TNF-α or pHAGE-GFP) and X-tremeGENE HP DNA transfection reagent (Roche, Mannheim, Germany). psPAX2 and pMD2G plasmids were gifts from Didier Trono (Addgene plasmid # 12260 and # 12259, respectively). The ratio of lentiviral DNA construct: psPAX2: pMD2G was 1:2:2 and the ratio of the DNA construct: XtremeGENE HP DNA transfection reagent was 1:3.5. Cells were incubated with DNAXtremeGENE mixture for 48 hr at 37°C, 5% CO_2._ Medium containing viral particles were collected through a 0.45 μM sterile filter PVDF membrane (Merck Millipore, Ireland) and stored at -80°C [32].

### Lentiviral transduction: generation of HaCaT-TNF-α

HaCaT cells were seeded at 1x10^5^ cells in 35 x 10 mm cell culture dishes and incubated 24 hr to achieve 50–60% confluency at 37°C and 5% CO_2_. Cells were treated with 1 mL of medium containing virus pHAGE-TNF-α viral particle mixed with 8 μg/mL of polybrene transfection reagent [32] (Merck Millipore, Darmstadt, Germany) and incubated at 37°C for 4 hr. After 4 hr, 1 mL of DMEM supplemented with 10% FBS was added to each well and cells were incubated with the viral particles for an additional 48 hr. Viral transduced HaCaT cells were grown in HaCaT medium supplemented with 700 μg/mL G418 (Gibco/Life Technologies, Gaithersburg, USA) for 14 days to select for HaCaT cells with inducible TNF-α (HaCaT-TNF-α). After 14 days, cells were trypsinized and cultured in DMEM supplemented with 350 μg/mL G418.

### Gel electrophoresis and Western blot analysis

Various concentrations of Dox (Merck Millipore, Darmstadt, Germany) were added to HaCaT-TNF-α cells to induce the expression of TNF-α proteins. TNF-α protein expression in cell models was detected by western blotting as previously described [33, 34]. Briefly, cells were washed once with ice-cold phosphate buffered saline (PBS; HyClone Laboratories, Logan, USA), and resuspended in RIPA lysis buffer (Merck Millipore, Billerica, USA) containing a proteinase inhibitor cocktail (Roche, Mannheim, Germany) on ice for 5 minutes. Cells were scraped and lyzed. Lysates were incubated on ice for 30 min and centrifuged at 8000 rpm at 4°C for 10 minutes. Total protein concentrations were determined by Bradford assay (Bio-Rad, Hercules, USA). 15 μg of protein from lysate cells was fractionated on a 12% SDS-PAGE and proteins transferred onto PVDF membranes. Blotted membranes were blocked with 5% blotting-grade Blocker (Bio-Rad, Hercules, USA) for 1 hr at RT and washed with TBST buffer (1M Tris pH 7.4, 5M NaCl, 0.05% Tween20, dH20). Blots were incubated with (1:1000 v/v) primary antibody recognizing TNF-α (6945 TNF-α specific rabbit monoclonal antibody [35]), or GAPDH antibody (1:10000 v/v) (Santa Cruz Biotechnology, CA, USA) overnight at 4°C. After incubation with primary antibodies, blots were washed three times with TBST buffer and incubated with secondary antibodies (1:10000 v/v) (anti rabbit IgG HRP linked Ab (Cell Signaling Technology, Danvers, USA)) for 1 hr at RT. Protein bands were visualized on X-Ray films by chemiluminescence reaction using Pierce® ECL Western Blotting Substrate (Thermo Scientific, Rockford, USA). Protein bands on X-Ray films were quantitated and analyzed using the Gel documentation system (Syngene, Frederick, MD, USA) and GeneTools software (Image Analysis Software).

### Subcellular fractionation and membrane preparation

Subcellular fractionation and isolation of the membrane-rich fraction was carried out by differential centrifugation as previously described [36]. Briefly, HaCaT-TNF-α cells were cultured in 100 mm plates until 70% confluent and treated with or without 1 μg/ml of Dox for 48 hr. Cells were washed twice with ice-cold PBS, pH 7.4 and SF buffer (250 mM Sucrose, 20 mM HEPES (pH7.4), 10 mM KCl, 1.5 mM MgCl_2_, 1 mM EDTA, 1 mM EGTA) containing protease inhibitor cocktail (Roche, Mannheim, Germany) added immediately and solution placed on ice. Cells were harvested by scraping with a cell scraper to collect cell lysate, and transferred to 1.5 ml tubes and rotated end-over-end at 4°C for 30 min. Lysates were centrifuged at 720 x g at 4°C for 5 min and the supernatant was transferred to a new 1.5 ml tube and centrifuged, 10,000 x g at 4°C for 10 min. The supernatant containing cytosolic and membrane fraction was transferred to a new 1.5 ml tube and centrifuged in an ultracentrifuge, 100,000 x g at 4°C for 1 hr before carefully transferring the supernatant, cytosolic fraction, to a new 1.5 ml tube. The pellet was washed and resuspended with SF buffer and centrifuged, 100,000 x g at 4°C for 1 hr. The supernatant was removed and the remaining membrane fraction pellet resuspended in RIPA buffer (Merck Millipore, Billerica, USA) containing protease inhibitor cocktail (Roche, Mannheim, Germany). Twenty micrograms of protein per sample was loaded onto 12% SDS-PAGE and analyzed by Western Blot as previously described. GAPDH was used as a loading control for cytosolic protein contamination.

### ELISA assays

Dox-induced TNF-α was secreted from HaCaT-TNF-α cell cultures and supernatants and analyzed after 24, 48, 72, and 96 hrs. TNF-α was measured by ELISA assays (#0509025 Peprotech, NJ, US) as recommended by the manufacturer. Briefly, the ELISA plate was coated with 1 μg/mL capture antibody (antigen-affinity purified rabbit anti-hTNF-α and 2.5mg D-mannitol) for 24 hr at RT. After 24 hr the plate was washed with wash buffer (0.05% Tween-20 in PBS) four times and then blocked with blocking buffer (1% BSA in PBS) for 1 hr RT. After blocking, the plate was washed with wash buffer. Human TNF-α standard was diluted to concentrations 0–2 ng/mL in diluent (0.05% Tween-20 in 0.1% BSA-PBS) and standards and samples were added to each well in triplicate for 2 hr at RT. The ELISA plate was washed four times and 0.5 μg/mL detection antibody (biotinylated antigen-affinity purified rabbit anti-hTNF-α + 2.5mg D-mannitol) added for 2 hr and washed four times. Avidin-HRP conjugate (1:2000) was added and incubated for 30 min and then washed four times. Finally, ABTS substrate was added into each well and color development monitored with an ELISA plate reader (Biotek Synergy Mx microplate reader, Biotek, USA) at 405 nm. The plate was monitored at 5 min intervals for approximately 30 min. For quantitative analysis a standard curve was generated for each assay using serially diluted hTNF-α protein standard concentration from 0–2 ng/mL for a total of 8 data points.

### Cell proliferation assays

HaCaT-TNF-α and–GFP cells were seeded in DMEM supplemented with 10% FBS and 1% PenStrep at 2,000 cells per well in a 96-well tissue culture plate and incubated for 24 hr to achieve 50–60% confluence. Next day, cells were induced with increasing concentration of Dox (0, 1, 2 and 3 ug/mL) for 24, 48, 72 and 96 hrs. In some cases, cells were treated with 10 ng/mL TNF-α for 24, 48, 72 and 96 hrs. Following treatments, 20 ul per well of 50 mg/mL MTT ((3-(4, 5-dimethylthiazolyl-2)-2,5-diphenyltetrazolium bromide) solution (Applichem, Darmstadt, Germany) were added to cells and incubated for 4 hr until purple precipitate was visible. Precipitates were dissolved with DMSO, centrifuged at 1000g for 10 min and were transferred to a new 96-well plate and measured using a microplate spectrophotometer (Biotek Synergy Mx microplate reader, Biotek, USA) absorbance 550 nm. All experiments were carried out in quadruplets and data was calculated from three independent experiments as mean ± SEM. Results are presented as % cell viability as compared to the control value of each experiment.

### Quantitative reverse transcription-PCR

Total RNA was extracted from HaCaT-TNF-α cell lines using Genezol reagent (GeneAid, Taiwan). cDNA was synthesized from 1 μg of total RNA using Accupower RT Premix (Bioneer, Korea) with oligo (dT) 18 primer following the manufacturer's protocol. Quantitative real-time PCR for IL-1β, IL-6, IL-8, NF-κB1, KRT16, FOSL1, MMP9 and GAPDH was performed by using Green Star PCR Master Mix (Bioneer, Korea) and gene-specific primers, as listed in Table 1, following the manufacturer’s instructions. The PCR for all genes were performed and fluorescent signals were measured in real time under the following conditions: 95°C for 10 min, 35 cycles at 95°C for 15 sec, 54°C for KRT16, 55°C for FOSL-1 and IL-1β, 57°C for GAPDH and MMP9, 58°C for IL-8, NF-κB1 and IL-6 for 30 sec. The fluorescent signal was expressed as the number of cycles required to achieve fluorescence at threshold. To normalize the amount of total RNA added to each reaction, GAPDH was used as an internal control and relative gene expressions were presented with the 2^-ΔΔCt^ method [37].

**Table 1:** Sequences of gene-specific primer pairs used in RT-qPCR.

Gene name	Sequences	PCR product size (bp)
GAPDH (control)	Forward 5’ ACA TCG CTC AGA CAC CAT G 3’	143
	Reverse 5’ TGT AGT TGA GGT CAA TGA AGG G 3’	
IL-8 [26, 50]	Forward 5’ CTG CGC CAA CAC AGA AAT TA 3’	238
	Reverse 5’ ATT GCA TCT GGC AAC CCT AC 3’	
NF-κB1	Forward 5’ GAA CCA CAC CCC TGC ATA TAG 3’	133
	Reverse 5’ GCA TTT TCC CAA GAG TCA TCC 3’	
IL-6	Forward 5’ CCA CTC ACC TCT TCA GAA CG 3’	150
	Reverse 5’ CAT CTT TGG AAG GTT CAG GTT G 3’	
IL-1β	Forward 5’ ATG CAC CTG TAC GAT CAC TG 3’	142
	Reverse 5’ ACA AAG GAC ATG GAG AAC ACC 3’	
MMP9	Forward 5’ CAG TTT CCA TTC ATC TTC CAA GG 3’	150
	Reverse 5’ CAT CAC CGT CGA GTC AGC 3’	
KRT16	Forward 5’ TGA ATG AGA TGC GTG ACC AG 3’	126
	Reverse 5’ TCT GTA CCA GTT CGC TGT TG 3’	
FOSL1	Forward 5’ GGG CAT GTT CCG AGA CTTC 3’	138
	Reverse 5’ CTC ATG GTG TTG ATG CTT GG 3’	

### Statistical analysis

Results were expressed as the mean ± standard error of means (S.E.M.) of at least three independent experiments. Differences between the control and variable conditions were determined by a two-tailed Student’s t-tests and one way ANOVA and results were considered to be significant at P < 0.05 or P < 0.01.

## Results

### Construction and characterization of tetracycline-inducible TNF-α expression in HaCaT cell models

To develop a novel *in vitro* TNF-α producing keratinocyte model, we constructed an *in vitro* cell model to study TNF-α function in HaCaT cells. HaCaT, an immortalize and spontaneously transformed human keratinocyte cell line [30], was chosen as a cell model for this study. HaCaT cell line is widely used as a model for various skin inflammation conditions such as psoriasis, UV-induced dermatitis or atopic dermatitis. In previous studies, HaCaT cells were treated with exogenous TNF-α and analyzed for pro-inflammatory responses [38–40]. In this study, we constructed HaCaT cells expressing inducible TNF-α by transducing cells with lentiviral constructs [31]. Human TNF-α cDNA (hTNF-α) was cloned into a lentiviral Tet-inducible vector as described in Materials and Methods. Tet-inducible expression vectors of TNF-α were designated HaCaT-TNF-α. As a control, HaCaT cells expressing inducible GFP, HaCaT-GFP, were similarly constructed as described in Materials and Methods. HaCaT cells expressing inducible TNF-α were then analyzed for biological responses to TNF-α and compared with those from HaCaT cells treated with exogenous TNF-α.

### Dox-induced TNF-α expression in HaCaT-TNF-α cells

**Fig 1:**
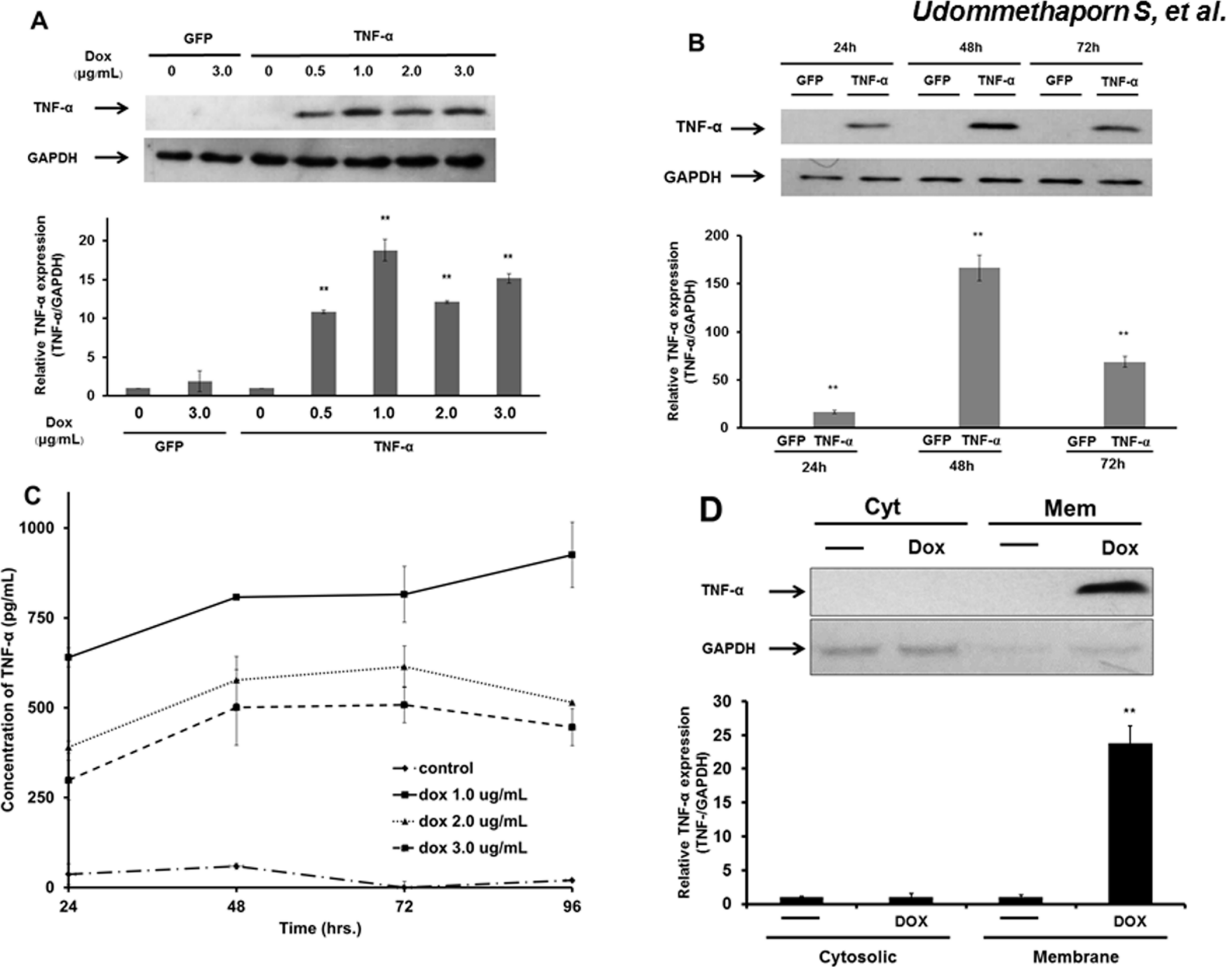
Effects of doxycycline on expression of TNF-α protein by Western blotting analysis and ELISA. (A) HaCaT-TNF-α (TNF-α) and HaCaT-GFP (GFP) cells were treated with increasing doxycycline (Dox) concentrations (0, 0.5, 1.0, 2.0, and 3.0 **μ**g/mL) for 48 hrs. Cells were lyzed and analyzed using Western blot with TNF-α-specific antibody rabbit monoclonal antibody. Fifteen micrograms of proteins were loaded on each lane. GAPDH was used as loading control. Bar graphs represent relative TNF-α expression normalized with GAPDH. (B) Same as (A) except HaCaT-TNF-α and HaCaT-GFP cells were treated with 1.0 **μ**g/ml Dox and incubated for 24, 48 and 72 hrs. (C) HaCaT-TNF-α were treated with increasing doses of Dox (1.0, 2.0, and 3.0 **μ**g/mL) and incubated for 24, 48 and 72 hrs to induce secretion of TNF-α proteins. The supernatants were prepared and detected by ELISA. Line graphs represent concentration of TNF-α secreted into the supernatant after treated with doxycycline. All data represent mean ± SEM from three independent experiments. (D) HaCaT-TNF-α were treated with and without 1.0 **μ**g/ml Dox and incubated for 48. Cells were harvested and subjected to subcellular fractionation into cytoplasmic (Cyt) and membrane (Mem) fractions. Fifteen micrograms of proteins were loaded on each lane. GAPDH was used as cytoplasmic markers and loading control. Bar graphs represent relative TNF-α expression normalized with GAPDH. ** (p< 0.01)

To test whether TNF-α protein was induced by Dox treatment, HaCaT-TNF-α and -GFP control cells were treated with increasing concentrations of Dox for 48 hr. Cells were harvested and analyzed for TNF-α expression by western blotting analysis using a TNF-α specific monoclonal antibody as described in Materials and Methods. Dox treatment (3 μg/mL) did not induce TNF-α expression in HaCaT-GFP cells (Fig 1A and 1B). However, Dox-treatment (0.5–3 μg/mL) dose-dependently increased TNF-α expression with maximum Dox concentration at 1 μg/mL (Fig 1A). To determine the optimal time for TNF-α induction, HaCaT-TNF-α and -GFP cells were treated with 1 μg/mL Dox for 24, 48 and 72 hrs. No TNF-α expression was detected at all time points in HaCaT-GFP cells. However, TNF-α expression was detected at 24 hr with maximum expression at 48 hr in HaCaT-TNF-α (Fig 1B). To examine whether the inducible TNF-α produced by the HaCaT cells was secreted into the growth medium, HaCaT-TNF-α cells were treated with increasing doses of Dox (1–3 μg/mL) and cultured for 24–96 hr. Conditioned medium at 24, 48, 72 and 96 hr after Dox-treatment was collected and analyzed for variability in TNF-α concentrations using an ELISA method described in Materials and Methods. As shown in Fig 1C, very little to no TNF-α was detected in condition medium collected from control (untreated) HaCaT-TNF-α cells. Dox treatment (1–3 μg/mL) induced TNF-α expression in HaCaT-TNF-α cells and resulted in an increase in TNF-α concentration over time, 24–96 hrs post Dox treatment (Fig 1C). TNF-α was detectable within 24 hr post Dox treatment and 1 μg/mL of Dox was found to be optimal to induce the highest concentrations of secreted TNF-α in HaCaT-TNF-α cells (Fig 1C).

Previous studies showed that newly synthesized TNF-α is expressed on plasma membranes and subsequently cleaved by TNF-α converting enzyme releasing soluble TNF-α proteins into the cell growth medium [9, 21]. To confirm the expression of TNF-α on plasma membranes, HaCaT-TNFα cells were treated with or without 1 μg/ml of Dox for 48 hr. Cells were lyzed and fractionated into cytosolic and membrane fraction using differential centrifugation methods as previously described [36]. Proteins from cytosolic and membrane fractions were subjected to Western Blotting analysis using GAPDH as the cytoplasmic loading control. No TNF-α expression was detected in either cytosolic or membrane fractions in untreated HaCaT-TNFα control cells. Dox treatment induced strong TNF-α protein expression that was detected in the membrane fraction but not in the cytosolic fraction (Fig 1D). Together, these data demonstrated that Dox induced HaCaT-TNF-α expressed both secreted and membrane bound TNF-α as demonstrated by ELISA membrane preparation and by Western blot analyzes of the membrane fraction, respectively.

### Effects of inducible TNF-α on keratinocyte cell viability

**Fig 2:**
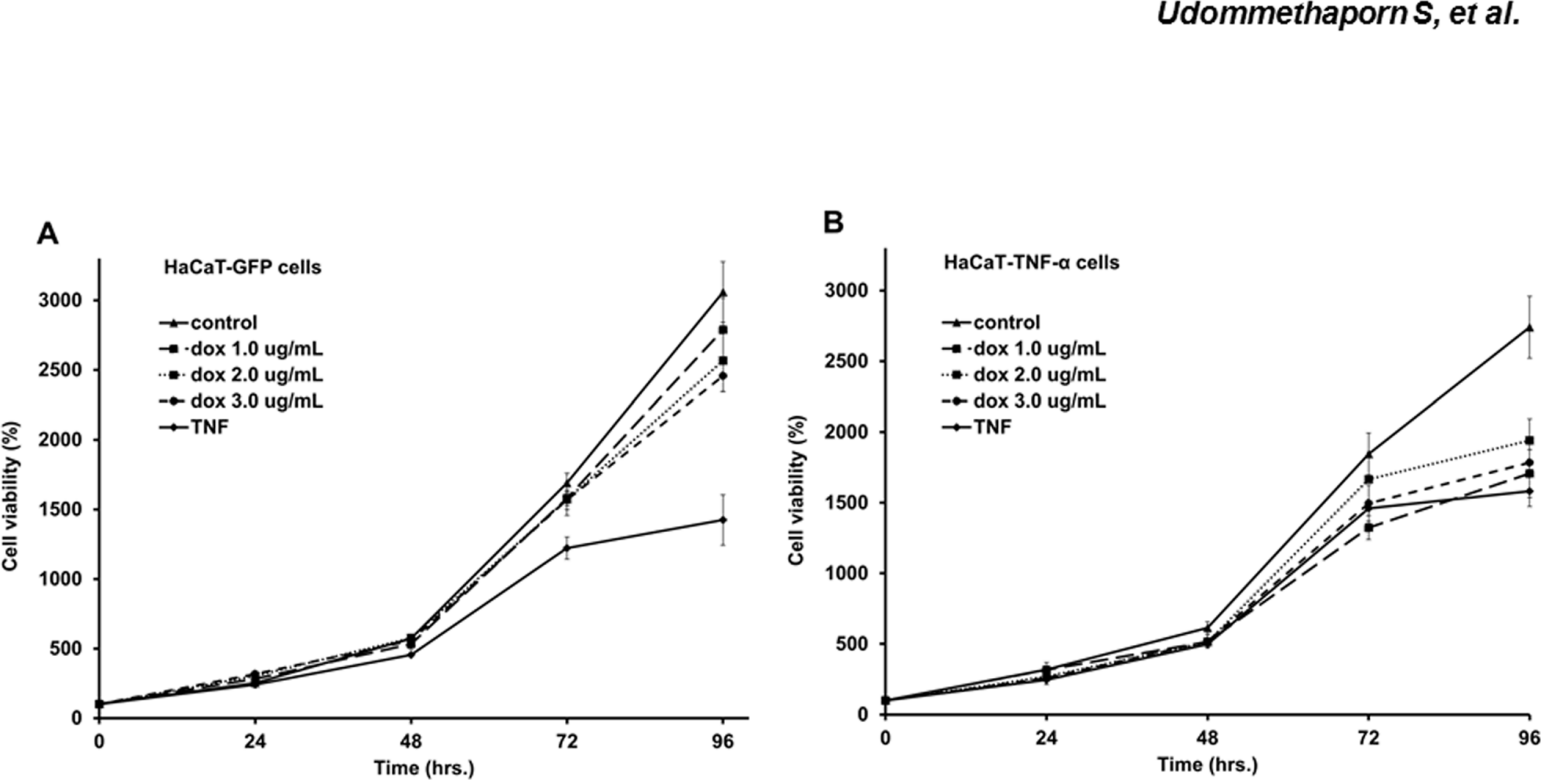
Effects of TNF-α expression on HaCaT cell viabilities. Line graphs represent percent cell viability of HaCaT-GFP (A) HaCaT-TNF-α (B) after treated with doxycycline (1.0, 2.0 and 3.0 **μ**g/mL) and 10 ng/mL TNF-α was used as a positive control. Values are represented as mean ± SEM (n = 3). (Percentage cell viability is shown as a percent of doxycycline-treated cells normalized with control untreated cells.)

To determine how Dox-induced TNF-α affected keratinocyte cell viability, HaCaT-GFP (control) and HaCaT-TNF-α cells were treated with increasing doses of Dox (1–3 μg/mL) or recombinant TNF-α (10 ng/mL) for 24–96 hrs. Percent cell viability was determined by MTT assays as described in Materials and Methods. In the control HaCaT-GFP cells TNF-α treatment significantly decreased cell viability (Fig 2A and 2B) whilst treatment with Dox had no effect at all doses tested. This confirmed that Dox and GFP had little to no effect on HaCaT-GFP cells (Fig 2A). Similarly, exogenous treatment of TNF-α in un-induced HaCaT-TNF-α cells significantly decreased cell viability (Fig 2A and 2B). Cell viability of HaCaT-TNF-α cells post induction of TNF-α with Dox treatment significantly decreased HaCaT-TNF-α cell viability at all doses tested: similar to treatment with 10 ng/mL recombinant TNF-α (Fig 2B). These experiments demonstrated that Dox-induced TNF-α expressed from HaCaT-TNF-α was biologically active mimicking exogenous TNF-α treatment.

### Effects of Dox on pro-inflammatory cytokine gene expressions in HaCaT-TNF-α cells

**Fig 3:**
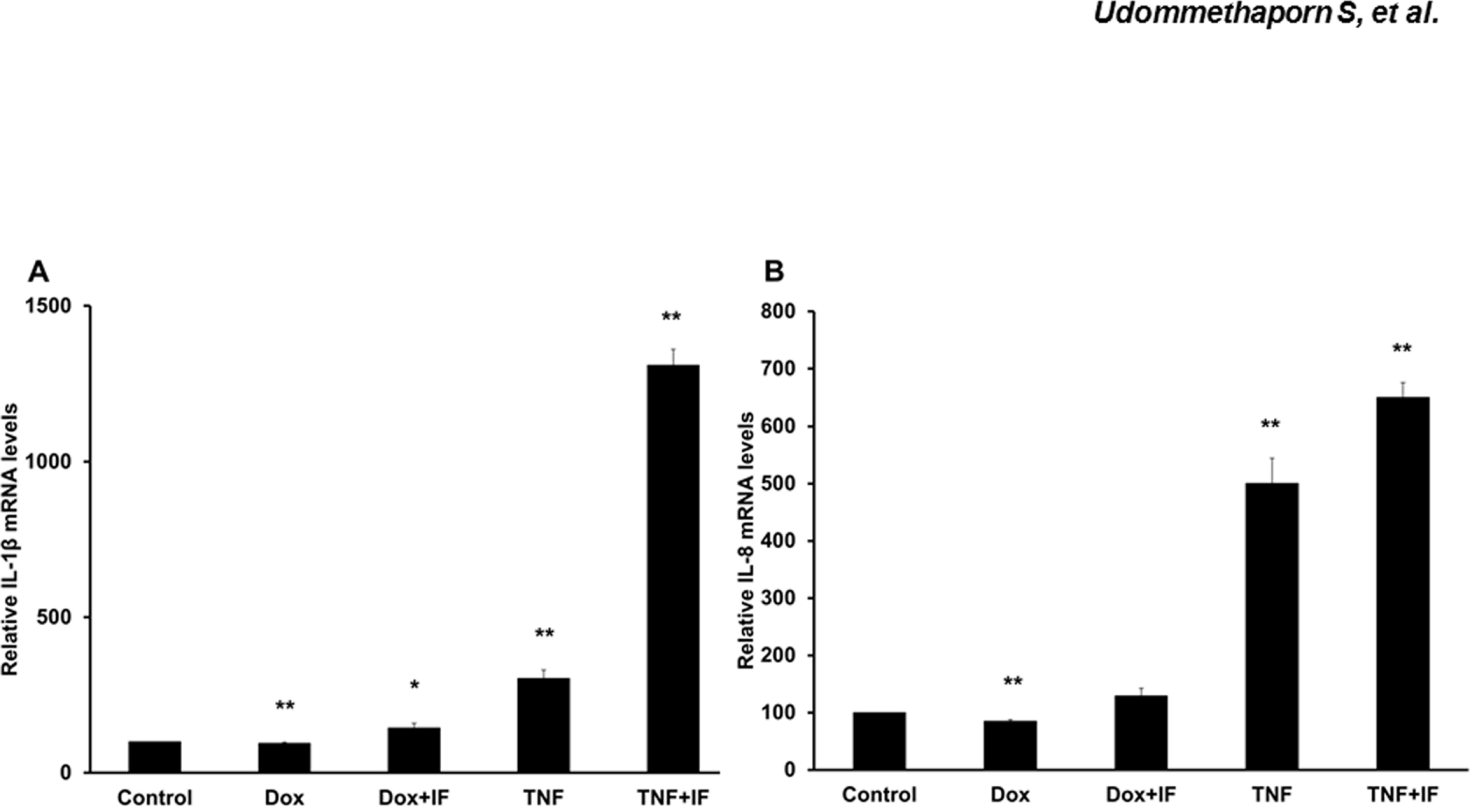
Effects of doxycycline induces pro-inflamatory cytokines (IL-1β and IL-8) expression in HaCaT cells. HaCaT cells were treated with 1.0 **μ**g/mL doxycycline (Dox) or 10 ng/mL TNF-α for 24 hrs. and then treated with or without 10 ng/mL IFN-γ for a further 24 hrs. Total RNA was extracted and reverse-transcribed. cDNA was amplified by qRT-PCR using IL-1β and IL-8 primers (Table 1). GAPDH was used as loading control. Bar graphs represent relative gene expression (A) IL-1β (B) IL-8 expression where values represent relative gene expression normalized with GAPDH. Mean ± SEM from three independent experiments. *(*p*<0.05), **(*p*<0.01) compared with control group.

**Fig 4:**
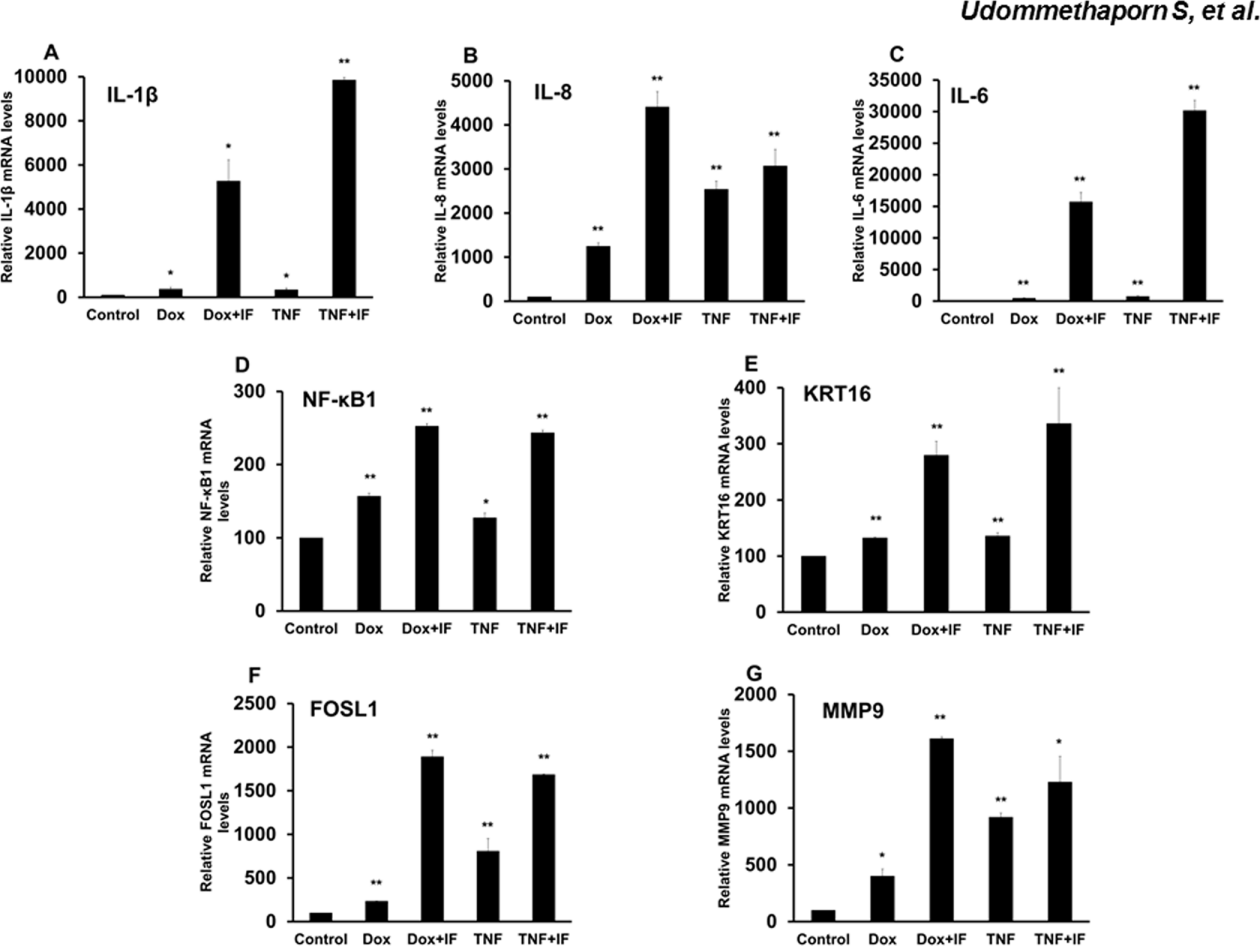
Effects of doxycycline induces pro-inflamatory cytokines (IL-1β, IL-8, IL-6, NF-κB1, KRT16, FOSL1 and MMP9) expression in HaCaT-TNF-α cells. HaCaT-TNF-α cells treated with 1.0 **μ**g/mL Dox or 10 ng/mL TNF-α for 24 hrs were then treated with or without 10 ng/mL IFN-γ for a further 24 hrs. Total RNA was extracted and reverse-transcribed. cDNA was amplified by qRT-PCR using IL-1β, IL-8, IL-6, NF-κB1, KRT16, FOSL1 and MMP9 primers (Table 1). GAPDH was used as loading control. Relative expression of IL-1β (A) IL-8 (B) IL-6 (C) NF-κB1 (D) KRT16 (E) FOSL1 (F) and MMP9 expression (G) are shown. Values represent relative gene expression normalized with GAPDH. Mean ± SEM from three independent experiments. *(*p*<0.05), **(*p*<0.01) compared with control group.

TNF-α promotes and maintains chronic inflammation conditions such as psoriasis through induction of several pro-inflammatory cytokines [11]. This prompted an investigation to whether Dox treatment induced pro-inflammatory cytokine genes. We first examined whether Dox had any effects on expression of pro-inflammatory cytokines IL-1β and IL-8 in HaCaT cells. Wild-type HaCaT cells were treated with Dox 1 μg/mL for 48 hrs. Cells were lyzed and mRNA was extracted and converted to cDNA following the manufacturer’s instructions. mRNA levels were quantitated by RT qPCR as described in Materials and Methods. Dox treatment failed to induce expression of IL-1β and IL-8 while TNF-α treatment significantly increased expression of IL-1β and IL-8 in HaCaT cells (Fig 3A and 3B), indicating that Dox treatment did not affect expression of pro-inflammatory cytokines, IL-1β and IL-8. We next examined whether Dox-induced TNF-α was biologically active and could stimulate expression of pro-inflammatory genes. HaCaT-TNF-α cells were treated with 1 μg/mL Dox or 10 ng/mL recombinant TNF-α for 48 hr. Cells were lyzed and mRNA levels of inflammatory mediators or pro-inflammatory cytokine genes including nuclear factor NF-kappa-B1 (NF-κB1), IL-6, IL-1β and IL-8 and pathogenic gene markers of psoriasis including keratin 16 (KRT16), Fos gene 1 (FOSL1) and matrix metalloproteinase 9 (MMP9) were determined by RT qPCR. In all gene tested, Dox-induced TNF-α and treatment with recombinant TNF-α resulted in similar induction of mRNA in HaCaT-TNF-α (Fig 4A–4G). Together, these data suggested that Dox treatment induced TNF-α that was biologically active and induced key TNF-α target genes and this effect was similar to exogenous treatment with recombinant TNF-α.

### Secreted and Membrane TNF-α from Dox-induced HaCaT-TNF-α cells stimulated pro-inflammatory cytokines expression in HaCaT cells

**Fig 5:**
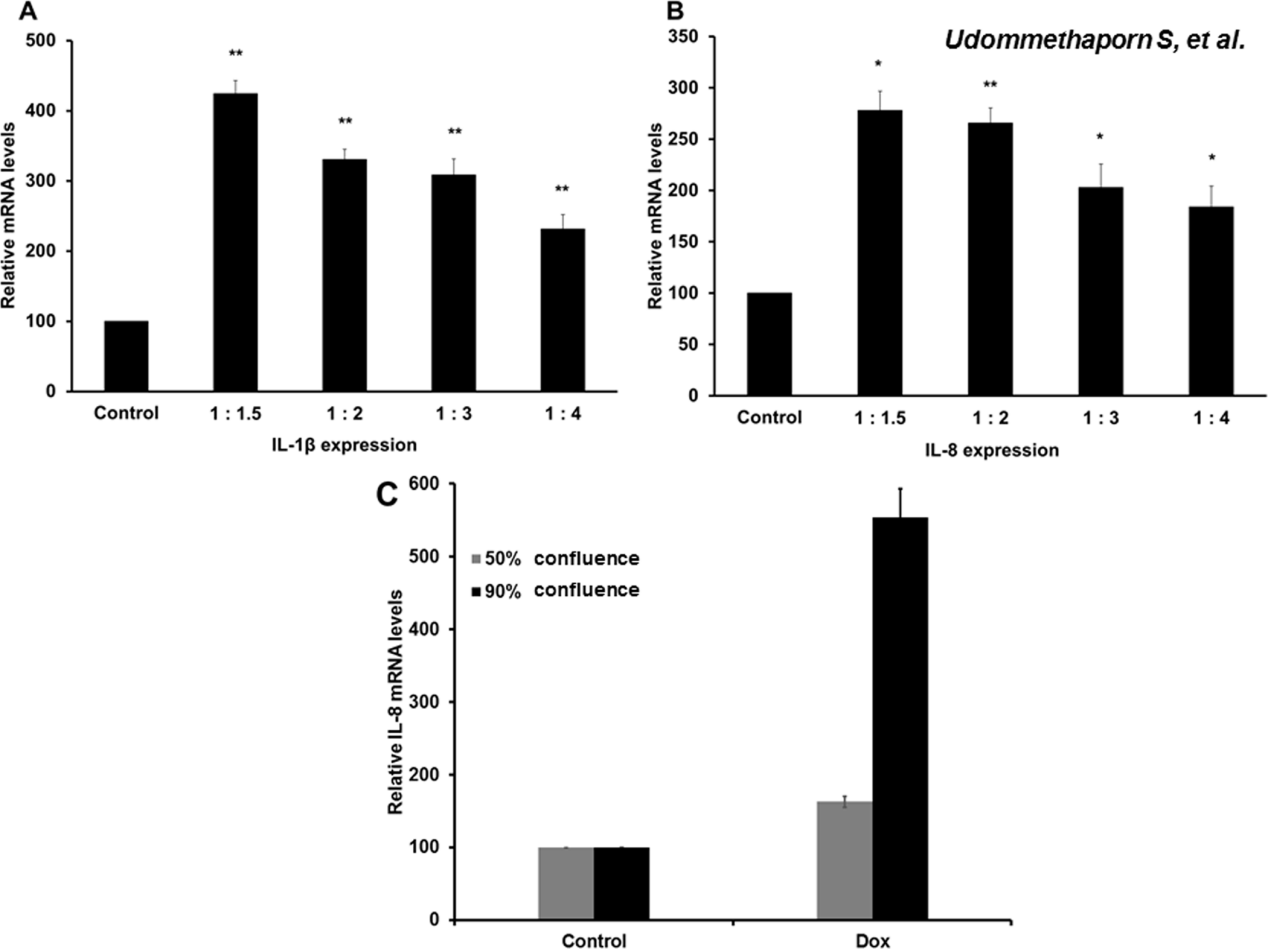
Effects of secreted TNF-α in supernatant stimulated pro-inflammatory cytokines (IL-1β and IL-8) expression and effects of membrane TNF-α on IL-8 expression in HaCaT cells. HaCaT-TNF-α cells were treated with 1.0 **μ**g/mL doxycycline (Dox) for 48 hrs. Supernatant collected was serially diluted with fresh medium in the ratio (supernatant fresh medium) 1:1.5, 1:2, 1:3 and 1:4. HaCaT cells were incubated with diluted supernatant for 24 hrs and subsequently treated with 10 ng/mL IFN-γ for a further 24 hrs. Expression levels of IL-1β and IL-8 were quantitated by qRT-PCR. Relative expression of IL-1β (A) and IL-8 (B) are shown. (C) HaCaT-TNF-α cells were cultured at different seeding densities and treated with 1 **μ**g/ml Dox for 48 hr. Cells harvested at approximately 50% or 90% confluence. IL-8 gene expression from cells at 50% and 90% confluency were analyzed by RT qPCR. Relative expression of IL-8 are shown. Results are represented as the Mean ± SEM from three independent experiments compared with control untreated *(*p*<0.05), **(*p*<0.01).

TNF-α is initially synthesized as pro-TNF-α, expressed on the plasma membrane and cleaved by ADAM-17 before releasing the soluble TNF-α protein [8, 23]. We found that HaCaT-TNF-α secreted TNF-α into the growth medium as measured by ELISA assay (Fig 1C). We next tested whether secreted-TNFα from Dox-induced HaCaT-TNF-α could stimulate expression of pro-inflammatory cytokine genes in wild-type HaCaT cells. HaCaT-TNF-α cells were treated with Dox 1 μg/mL for 48 hr. Growth medium was collected and subjected to centrifugation to remove cell debris. The Dox-induced supernatants were diluted with fresh growth medium with dilution ratios of supernatant to fresh medium of 1:1.5, 1:2, 1:3 and 1:4. Diluted Dox-induced supernatants were then transferred and incubated with wild-type HaCaT cells for 24 hr and treated with 10 ng/mL IFN-γ for an additional 24 hr. mRNA was extracted and expression of pro-inflammatory cytokine genes, IL-1β and IL-8, were detected by RT qPCR. The secreted TNF-α from Dox-induced HaCaT-TNF-α was able to induce IL-1β and IL-8 expressions in wild-type HaCaT cells in all dilutions tested (Fig 5A & 5B), indicating the secreted TNF-α in the Dox-induced conditioned medium was biologically active and could effectively be used as a source of active TNF-α to stimulate expressions of pro-inflammatory cytokines expressions in neighbouring cells.

In addition to producing secreted soluble TNF-α as shown in Fig 5A & 5B, HaCaT-TNF-α expressed Dox-induced membrane TNF-α as shown in Fig 1D. Previous studies suggested that both membrane bound and secreted TNF-α are biologically active [21]. To determine the involvement of membrane bound TNF-α in TNF-α mediated gene expression, HaCaT-TNF-α cells were cultured at different seeding densities and treated with 1 μg/ml Dox for 48 hr. Cells harvested at approximately 50% confluence exhibited little cell-cell contact, and cells harvested at 90% confluence exhibited extensive cell-cell contact. IL-8 gene expression from cells at 50% and 90% confluency were analyzed by RT qPCR. Dox treatment significantly induced IL-8 gene expression. However, Dox treatment of HaCaT-TNF-α cells at 90% confluency induced significantly better IL-8 gene expression as compared to cells at 50% confluency (Fig 5C). These data suggest that Dox-induced TNF-α in the HaCaT-TNF-α model is likely to involve TNF-α signaling through both juxtacrine cell signaling, via membrane-bound TNF-α, and paracrine/autocrine cell signaling, through secreted TNF-α. Dual actions of both membrane-bound and secreted forms of TNF-α are needed for maximum TNF-α induced gene expression.

### Using the HaCaT-TNF-α cell model to access compounds with anti-TNF-α activity

**Fig 6:**
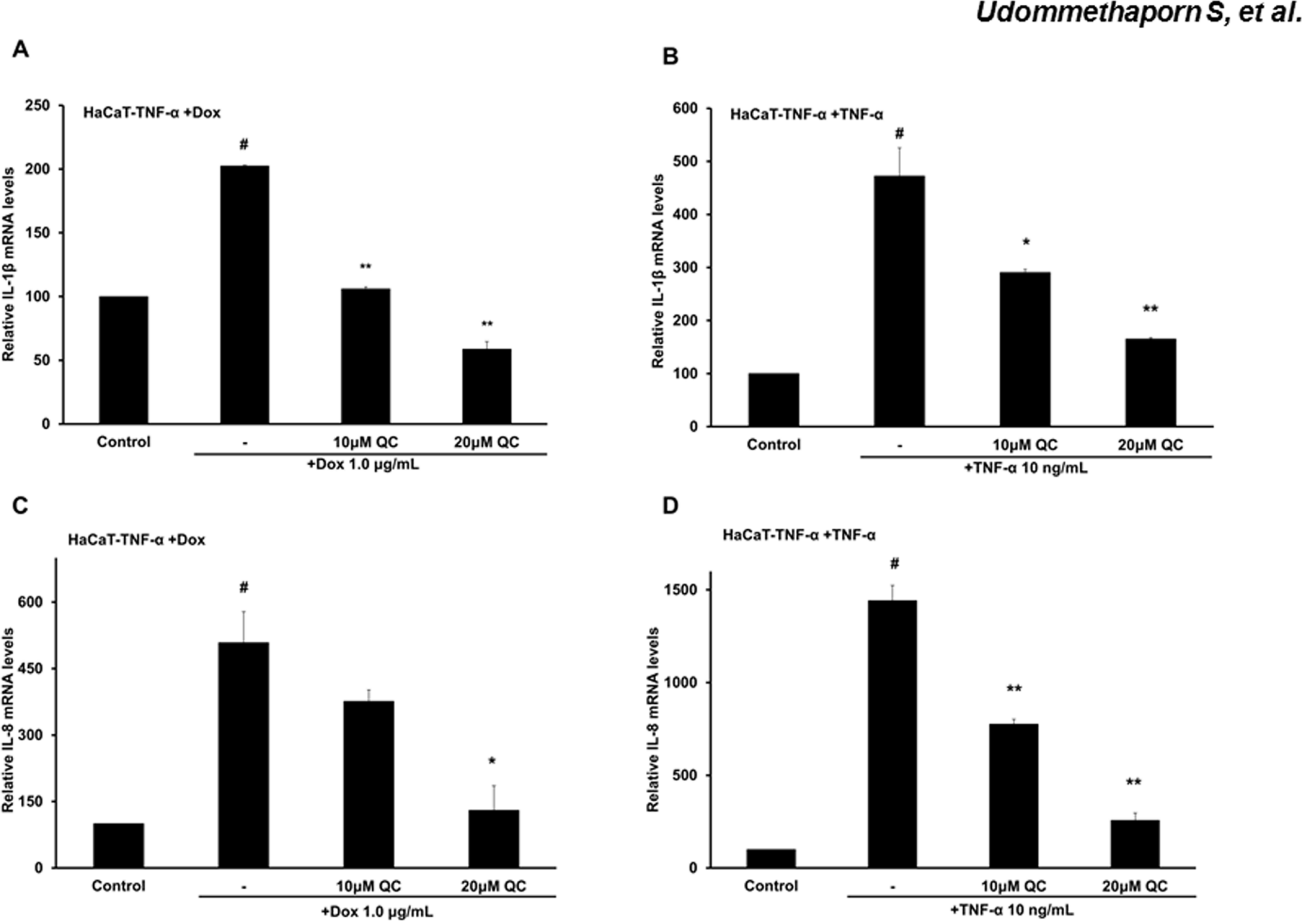
Quercetin inhibits pro-inflammatory cytokine (IL-1β and IL-8) expression. HaCaT-TNF-α cells pre-treated with 1.0 **μ**g/mL Dox or 10 ng/mL TNF-α for 24 hrs. Cells were then treated with 10 **μ**M and 20 **μ**M Quercetin for an additional 24 hrs. Total RNA was extracted and reverse-transcribed. cDNA was amplified by qRT-PCR. Relative expression of IL-1β (A-B) and IL-8 expression (C-D) are shown. Results are represented as Mean ± SEM from three independent experiments compared with control # (*p*<0.01) and compared with Dox or TNF-α treated *(*p*<0.05),**(*p*<0.01).

We next examined whether the HaCaT-TNF-α cell model could be used to assess the anti-TNF-α activity of a chemical compound Quercetin. Quercetin is a flavonoid that can be found in several types of fruits, vegetables, leaves and grains and has been shown to possess anti-psoriatic and anti-inflammatory activities [39, 41] To determine anti-TNF-α activity, we examined Quercetin’s ability to reduce expressions of pro-inflammatory cytokine genes, IL-8 and IL-1β, stimulated by Dox-induced TNF-α or recombinant TNF-α in HaCaT-TNF-α cells. HaCaT-TNF-α cells were treated with Dox 1 μg/mL or 10 ng recombinant TNF-α for 24 hr before treating with 10 or 20 μM Quercetin for an additional 24 hr. mRNA were extracted and expressions of IL-1β and IL-8 were quantitated by RT-qPCR. Treatment with Quercetin, an anti-TNF-α compound, dose-dependently decreased expression of IL-8 and IL-1β (Fig 6). Together, these data suggested that the Dox-induced TNF-α could be used in place of recombinant TNF-α for treatment of HaCaT-TNF-α for screening of compounds with anti-TNF-α activities.

## Discussion

Dysregulation or untimely inflammation can lead to many diseases such as Alzheimer’s, cancer cardiovascular, arthritis, inflammatory bowel diseases, and psoriasis [1, 5]. TNF-α is a major pro-inflammatory cytokine that plays a crucial function in regulating the intricate network of cytokines in different tissues. However, most *in vitro* models used to study TNF-α and anti-TNF-α activities involve treating cells with exogenous recombinant TNF-α and examining changes associated with the TNF-α treatment [26–29]. While exogenous TNF-α treatment mimics many pathophysiological conditions observed in inflammatory diseases, treating cells with exogenous TNF-α only acts on one arm of TNF-α function, as a secreted or cleaved TNF-α affecting TNF receptors. This action does not affect perhaps an equally important role in cell-cell dependent signaling mediated by membrane-bound TNF-α. While the role of bound and secreted TNF-α signaling is not clear, previous studies suggested that different forms of the TNF-α molecule have the potential to elicit different biological responses [21, 42]. The presence of dual TNF-α signaling from both bound and free forms is likely to affect analysis of the overall response to TNF-α or anti-TNF-α.

To develop a new and improved model to investigate TNF-α and anti-TNF-α activities that would enable examination of TNF-α dual signaling from both membrane-associated and cleaved TNF-α, we took advantage of lentiviral technology and constructed epidermal keratinocytes (HaCaT cells) [30] expressing inducible TNF-α, HaCaT-TNF-α. Using Western blot analysis we demonstrated expression of cellular TNF-α could be readily induced by addition of Dox (1 μg/mL) with maximal induction at 48 hr post Dox treatment. Determination of Dox-induced TNF-α in culture medium using ELISA assays confirmed the presence of physiologically relevant levels of soluble TNF-α ranging from 0.3–0.9 ng/mL similar to TNF-α levels in extracts from lesional psoriatic stratum cornuem [43]. The levels of TNF-α from psoriatic extracts and the levels of secreted TNF-α in our cell model (0.3–0.9 ng/mL) were about ten-fold lower than the concentration of exogenous TNF-α (10 ng/mL) used in the current *in vitro* cell models [26–29]. Comparison of cells treated with exogenous TNF-α and cells expressing inducible TNF-α in this study revealed similar TNF-α-mediated responses with respect to cell inhibition and induction of pro-inflammatory cytokines. However, these TNF-α-mediated responses could be achieved at a much lower TNF-α concentration in the HaCaT-TNF-α cell model as compared to HaCaT treated with exogenous TNF-α. These data suggest that cells expressing inducible TNF-α used in our model are more sensitive to the secreted TNF-α than cells treated with exogenous TNF-α. This could be due to cells expressing inducible TNF-α have dual signaling from secreted and membrane-bound forms of TNF-α, while cells treated with exogenous TNF-α have the majority of TNF-α signaling generated from secreted form of TNF-α.

How TNF-α mediated cell signaling in keratinocytes is unclear. A previous study showed that TNF-α upregulated its own mRNA through an autocrine mechanism but soluble TNF-α was undetected in the conditioned medium. Autocrine upregulated TNF-α mRNA levels declined sharply upon removal of TNF-α [44]. Membrane-bound TNF-α was moderately induced when keratinocytes were treated with a TNFR1 agonistic antibody (HTR9) for 8 hours [44]. Whether TNF-α treatment increases membrane-bound TNF-α is not known. It remains unclear whether cells treated with soluble exogenous TNF-α would signal similarly to cells producing TNF-α themselves. Thus, in certain pathological conditions where cells themselves express and produce TNF-α, this Dox-induced TNF-α cell model would be more physiological relevant than other *in vitro* cell models that use only exogenous TNF-α to activate TNF-α signaling.

In addition to inflammation, keratinocyte hyperproliferation and abnormal differentiation are often observed in psoriatic skin lesions [45, 46]. However, several studies have demonstrated TNF-α treatment or exogenous TNF-α expression in normal keratinocytes result in an increase in apoptosis and a decrease in cell proliferation [47–49]. Previous studies showed that addition of TNF-α dose dependently inhibited cell proliferation and induced apoptosis in HaCaT human keratinocyte cell line [29]. In this study, we showed that Dox-induced expression of TNF-α also dose dependently inhibited HaCaT-TNFα cell proliferation (Figs 1 and 2). Dox, itself, had no effect on expression and secretion of TNF-α in wild-type HaCaT cells (Fig 1A–1C). Using this inducible TNF-α cell model, we showed that Dox-induced TNF-α in HaCaT-TNF-α was biologically active and was able to induce expression of several TNF-α-mediated pro-inflammatory genes, including IL-1β, IL-8, NF-κB1, FOSL1, MMP9 and KRT-16 (Fig 4A–4G). It is interesting to note that cells treated with Dox-induced TNF-α or recombinant TNF-α gave similar, but not identical fold-activations (Fig 4). These differences in fold of gene activations could due to differences in soluble TNF-α concentrations present in culture medium or differences in how TNF-α signals to activate different genes. While the Dox-induced secreted TNF- α were present in small quantities, the amount of the secreted TNF-α was sufficient and transferable to induce expression of key pro-inflammatory cytokines, IL-1β and IL-8 of wild-type HaCaT cells (Fig 5A and 5B), suggesting that the HaCaT-TNF-α cell model could be used as a source for production of soluble and biologically active TNF-α. In addition, pro-inflammatory cytokine gene expression (IL-1β and IL-8) induced by exogenous TNF-α and Dox-induced TNF-α was similarly blocked by an anti-TNF-α compound, Quercetin (Fig 6A and 6B). These data demonstrated that our newly developed HaCaT-TNF-α cell model could be used to assess anti-TNF-α activities and screen for anti-TNF-α compounds.

We propose that this newly developed HaCaT-TNF-α cell model, whereby expression of TNF-α can be controlled by variable Dox concentrations, can be used as an alternative or a complementary cell system to the current *in vitro* cell models. Furthermore, this inducible HaCaT-TNF-α lentivirus system can be applied to any cell model, is not confined to one specific cell line. Therefore, an important application for this system, inducible TNF-α can be used to further investigate the dual cell signaling pathways in different inflammatory disease states. A future application for Dox-inducible TNF-α model is the analyzes of TNF-α activities and screening of small molecule compounds for anti-TNF-α activities: providing an effective, fast, and simple *in vitro* screening for compounds with anti-TNF-α activities for chronic inflammatory disease therapies.
